# Incorporating Diversity, Equity, and Inclusion Into the History of the Japan Primary Care Association

**DOI:** 10.1002/jgf2.70174

**Published:** 2026-09-08

**Authors:** Makiko Ozaki

**Affiliations:** ^1^ Murasakino Kyouritsu Clinic Kyoto Japan

To the Editor,

I read with great interest the special article by Matsumura et al., “The Formation and Evolution of the Japan Primary Care Association: A 15‐Year Retrospective” [1]. The article provides a valuable overview of the Japan Primary Care Association (JPCA), including its merger of three predecessor societies, certification systems, disaster response, infectious disease activities, academic awards, and interprofessional training. I appreciate this effort to document JPCA's first 15 years and its contributions to primary care in Japan.

This account, however, would be more complete if it incorporated a diversity, equity, and inclusion (DE&I) perspective. No women physicians are mentioned by name, and the contributions of women and inter‐professional members receive limited attention. This raises a broader question about how institutional histories are written, whose contributions are recognized, and whose work remains unnamed in narratives of professional development.

Professional societies shape education, research, clinical practice, leadership, visibility, legitimacy, and career opportunities. Historical accounts of such societies therefore shape which contributions are remembered as institution‐building. When retrospectives focus on formal structures, certification systems, named leaders, awards, and institutional milestones, the contributions of women physicians and inter‐professional members may become less visible, especially when they involve education, committee work, conference organization, community‐based practice, collaboration, and DE&I‐related initiatives. Such contributions are essential to primary care but may be underrecognized in conventional institutional narratives.

JPCA's 2023 amendment to its officer selection rules is relevant in this regard. Approved at the General Assembly on June 11, 2023, the reform introduced a quota system for board elections [2]. The related announcement also noted the inclusion of women and inter‐professional members on the election management committee and emphasized the need to increase their representation among delegates. Including this reform would provide a fuller account of JPCA's efforts to enhance diversity in decision‐making.

This issue is important in Japanese medicine, where women physicians remain underrepresented in leadership positions within professional medical societies. Across 19 Japanese professional medical societies, women accounted for only 7% of board members, despite comprising nearly one‐quarter of the physician workforce [3]. These disparities highlight the historical significance of JPCA's efforts to improve representation in governance and the importance of documenting them as part of the association's development.

Future historical accounts could examine whether representation within JPCA has changed over time across membership, board membership, delegates, committees, conference leadership, invited speakers, awardees, research grant recipients, journal editors, and certification candidates. They could also consider how different forms of contribution have been recognized. Such analyzes would clarify whether JPCA's development has been accompanied by broader and more equitable participation in leadership, academic recognition, and decision‐making.

Incorporating a DE&I perspective would complement, rather than contradict, the institutional history presented by Matsumura et al. It would make JPCA's 15‐year history more complete by situating organizational growth within questions of access, representation, visibility, and accountability, thereby strengthening JPCA's role as a leading academic organization for primary care in Japan.

## Author Contributions

**Makiko Ozaki:** conceptualization, writing – review and editing, writing – original draft.

## Disclosure

The author is a member of JPCA's Diversity, Equity, and Inclusion Promotion Committee. The views expressed are the author's own and do not represent the committee or JPCA.

## Conflicts of Interest

The author declares no conflicts of interest.

## Data Availability

Data sharing not applicable to this article as no datasets were generated or analysed during the current study.
